# Recent Advances in ASIC Development for Enhanced Performance M-Sequence UWB Systems

**DOI:** 10.3390/s20174812

**Published:** 2020-08-26

**Authors:** Pavol Galajda, Martin Pecovsky, Miroslav Sokol, Martin Kmec, Dusan Kocur

**Affiliations:** 1 Department of Electronics and Multimedia Telecommunications, Faculty of Electrical Engineering and Informatics, Technical University of Košice, 042 00 Košice, Slovakia; martin.pecovsky@tuke.sk (M.P.); miroslav.sokol@tuke.sk (M.S.); dusan.kocur@tuke.sk (D.K.); 2 Ilmsens GmbH, 98693 Ilmenau, Germany; martin.kmec@ilmsens.com

**Keywords:** ASIC, UWB, M-sequence generator, SoC, active filter, miniature antenna

## Abstract

Short-range ultra-wideband (UWB) radar sensors belong to very promising sensing techniques that have received vast attention recently. The M-sequence UWB sensing techniques for radio detection and ranging feature several advantages over the other short-range radars, inter alia superior integration capabilities. The prerequisite to investigate their capabilities in real scenarios is the existence of physically available hardware, i.e., particular functional system blocks. In this paper, we present three novel blocks of M-sequence UWB radars exploiting application-specific integrated circuit (ASIC) technology. These are the integrated 15th-order M-sequence radar transceiver on one chip, experimental active Electronic Communication Committee (ECC) bandpass filter, and miniature transmitting UWB antenna with an integrated amplifier. All these are custom designs intended for the enhancement of capabilities of an M-sequence-based system family for new UWB short-range sensing applications. The design approaches and verification of the manufactured prototypes by measurements of the realized circuits are presented in this paper. The fine balance on technology capabilities (Fc of roughly 120 GHz) and thoughtful design process of the proposed blocks is the first step toward remarkably minimized devices, e.g., as System on Chip designs, which apparently allow broadening the range of new applications.

## 1. Introduction

It can be said, “for the right decision, the right information is necessary”. In today’s society, many critical decisions must be made on a daily basis to prevent either small personal or huge international disasters from happening, such as for example in industrial, transportation, or military segments of the global society in which we live. To obtain the proper information in an appropriate amount of time and with sufficient reliability, not only communication networks are needed, but a large variety of sensors are needed as well. One of the sensor concepts, which can be exploited to obtain valuable information about the scene under test, is the concept of ultra-wideband sensors e.g., for ranging and detection or material investigations (such as impedance spectroscopy). Thanks to their large bandwidth in the lower corner in the electromagnetic spectrum, they allow us to gather detailed information about the outer surface of objects, as well as gain insight into their internal structure. Thus, these short-range radar sensors may have an important role in the development of intensively discussed concepts called the Internet of Things (IoT) and Industry 4.0, where the observation of the surroundings or monitoring of the production processes will be essential for making the “right decisions”.

The development of M-sequence ultra-wideband (UWB) radars has a long tradition in our department. In cooperation with TU Ilmenau, TU Ilmenau Service GmbH, and Ilmsens, GmbH, we have been a part of the exciting research in areas of data processing software as well as ultra-wideband hardware, enabling the M-sequence technology to take hold for short-range sensing. With continuous research and consequential technological improvements, new applications of the M-sequence radars are emerging, albeit with the perquisite named “minimal physical dimensions”. Despite successful integration of the core circuits of the radar, certain subcircuits of the M-sequence UWB radars are still made of discrete components. These include mainly the analogue front-end circuits. Antenna size is an issue as well, because the basic half-wave antenna approach that has been valid since the beginnings of radio has not been outperformed, even in modern systems. Thus, it limits the options for antenna miniaturization, especially at lower frequencies. So, the story of continuous miniaturization and performance progress, which is truly well rooted in the electronics over the past few decades, is indeed present in the field of ultra-wideband systems as well. This progress allows the realization of novel, more complex, high-performance sensing units as well as applications of new, sophisticated algorithms for gathered signals, enabling major improvements in detections, tracking, and imaging capability. To close up, this brings optimism toward new interesting UWB system realization perspectives, and thus, it is indisputable that the interest in (active) ultra-wideband systems has been rapidly increasing over the last few decades. This growing demand is for at least two reasons.

Firstly, it is due to UWB technology utilization in a broad scale of new promising sensing and communication applications [1]. So, for example, typical UWB sensing implementations include non-destructive testing tasks in industrial and civil engineering [2], non-invasive diagnostic and vital sign detection in medical engineering, as well as near-field microwave imaging or organ motion tracking [3,4], indoor localization, search and rescue or security tasks [5,6], and many others listed in next paragraph. By the same token, in communications, significant research concerning UWB technology has been invested, mainly focusing on high data rate (>100 Mb/s) short-range applications such as wireless multimedia and high-performance PC peripherals [7]. However, another relatively overlooked class of communication applications that make use of UWB’s unique low probability of detection, low interference, and multipath immunity is that of low data rates (<1 Mb/s) and very low power applications combined with new sensing and tracking capabilities [7,8], and so on.

The second and non-negligible UWB technology pushing element is the aforementioned omnipresent story of continuous miniaturization that had been predicted in the 1970s by Gordon Moore [9] and accompanied by exceeding technological achievements, resulting in rapid electronics performance upgrowth. So, for example, several decades after Moore’s predication, the physical gate length of elementary Metal-Oxide-Silicon (MOS) devices reach only several of nm [10], or enhanced vertical compositions of heterojunction bipolar transistors (HBTs) allow applications up to nearly the THz range [10,11]. As the result, the UWB system architects and designers of elementary functional blocks are freer to combine more units into complex networks (e.g., multiple inputs and multiple outputs systems, MIMO systems), or to put more functions in one device in order to realize them with unique (better) performance into less space, and do so for less cost per function (e.g., single chip devices). So, hand-in-hand with the technological efforts, the new UWB systems have real potential to satisfy the general need for higher system performance, i.e., higher operating speeds, higher overall system efficiency, improved dynamic behavior, higher complexity, and so on, and all this in a small form factor with fascinating performance e.g., [12,13]. Note that these are also essential prerequisites for the broader “market placement” of new UWB systems.

Regardless of an evident fact that the scene of UWB systems is indeed very broad, the scope of this work is most notably bounded to the UWB technology for short-range active sensing based on the so-called M-sequence approach [14,15]. The approach beneficially deploys a unique combination of high-frequency maximum length binary sequences (M-sequences) for stimulation of medium under test (MUT) and smart receiver architecture for capturing and processing of the signal interacted with the MUT. More detailed characteristics will be introduced in the next sections, and the interested reader can refer to [15] or [16] for more comprehensive understanding.

In the meantime, gathered experience and achieved system performance with such M-Sequence-based sensing instruments [17] show that these novel devices represent an interesting alternative to the classical sine or impulse-based UWB sensing systems. Exceptionally, the devices excel in terms of stability [18], facile extendibility [19], and ultra-compact realization perspective [20]. All of the characteristics make the M-Sequence devices viable candidates for a broad range of UWB sensing tasks and bring optimism to all involved parties. It is obvious that the development of new sensing systems is an interlaced task, which requires a broad scale of specialists. This scale normally starts with system architects, goes through designers of particular customized integrated circuits (ICs) or sophisticated printed circuit boards (PCBs), and terminates with processing algorithm programmers, application researchers, and finally the users.

In this work, we will try to point out possible first steps to cope with the difficulties stated above, based on the development of new application-specific integrated circuits (ASICs). First of all, we will propose a novel 15th-order M-sequence UWB radar system on chip (SoC), which includes all high-frequency blocks necessary for the basic M-sequence radar operation on a single die. Further, we will propose an active bandpass filter ASIC designed for operation with an M-sequence radar in the ECC band. The filter design is followed by the design of a miniature transmitting antenna for material probing applications, whose design is based on a customized ASIC amplifier as well. The proposed application-specific integrated circuits are technology-compatible with an M-sequence radar core chip, which is tested in real deployment for the first time in this work. The measurement results of the proposed radar components seem to be promising and, as usual, they raise new tasks for further research in this field.

## 2. M-Sequence UWB Radar Principles and Applications

For a given signal power, the M-sequence (maximum length binary sequence) is the signal that has the lowest amplitude [21]. Furthermore, it has the shortest autocorrelation function of all pseudo-random codes [15]. The short autocorrelation function makes the M-sequences ideal for the measurement of impulse response, which is the main task in the radar sensor technology. On the other hand, the mutual correlation of two M-sequences is non-zero; therefore, they may be susceptible to mutual interference in case of multi-channel excitation. However, this was proved to not be a problem if the M-sequence generators are operated from separate clock sources [22].

The M-sequence is defined as a pseudo-random sequence generated by a linear generator consisting of an *n*-bit shift register with appropriate feedback, whereas its period is *N* = 2*^m^* − 1 chips [22], where *m* is the order of the M-sequence.

**Figure 1:**
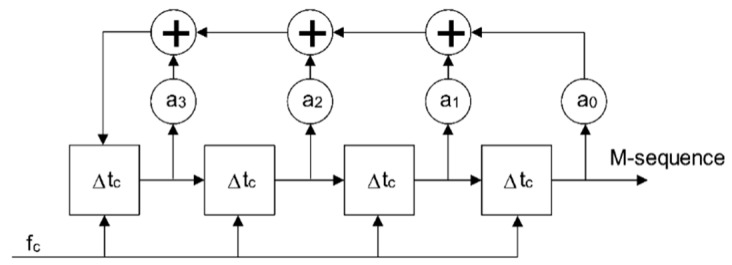
M-sequence generator example for 4th-order M-sequence.

The most straightforward way to generate the M-sequence is to use a digital shift register with feedback connections to modulo 2 adders (XOR gates), as shown in Figure 1. The shift register flip-flops are triggered by a clock signal at frequency *f_c_ =* 1*/t_c_*, where *t_c_* is duration of a single chip of the M-sequence. The feedback transmission coefficients *a_n_* have binary values 0 or 1. They must be chosen properly; otherwise, the generator does not work.

**Figure 2:**
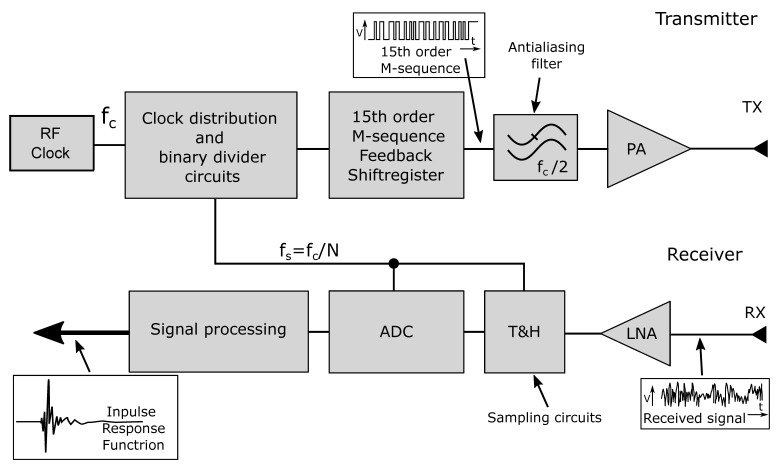
Block diagram of the M-sequence radar.

The basic block diagram of the M-sequence radar sensor is shown in Figure 2 [23]. The transmitting branch of the M-sequence radar is very straightforward—it includes the M-sequence generator described above, which is driven by a stable clock source. The clock stability is crucial for the precision of the sensor, whereas its frequency defines the maximum bandwidth, unambiguous range, and depth of material penetration if the sensor is used for material testing. Then, the bandwidth of the M-sequence signal from the generator is limited to the *f_c_/*2 band, which contains more than 70% of the signal energy. The resulting signal is amplified and transmitted by an antenna or appropriate probing interface.

**Figure 3:**
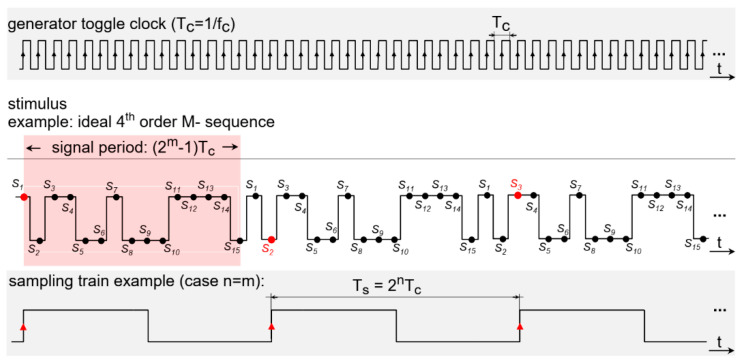
The timing diagram example of the equivalent time sampling for a 4th-order M-sequence.

The received signal is amplified again and fed into the ultra-fast Track & Hold (T&H) circuit. Here, the periodicity of the M-sequence signal is exploited, allowing the use of relatively cheap analog-to-digital converters (ADCs). The received signal is sampled at a frequency derived from the transmitter clock source by dividing it by a factor of *2^n^*, where *n* is a natural number. Common dividing factors are in the range from 32 to 256, and they dependent on *f_c_* and the measurement speed required, which allows the implementation of universal structures for the vast range of applications listed below. This way, only a few samples are acquired by the T&H circuit and digitized by ADC during each M-sequence period, whereas the whole impulse response is measured across multiple M-sequence periods employing so-called equivalent-time sampling. The timing diagram example of the equivalent-time sampling for an ideal 4th-order M-sequence is shown in Figure 3. The samples are digitally reordered and processed by correlation algorithms to form the impulse response (IR).

The M-sequence technology has multiple inherent properties that are well suited for measurement and sensing applications. Here, we will shortly emphasize several functions that are promoted by the M-sequence radars [15]:

- Small target detection and localization,
- Moving target detection,
- Target identification,
- Separation of stationary targets,
- Detection of hidden targets and investigation of opaque structures,
- Measurements of electrical and non-electrical material properties, e.g., by wideband impedance spectroscopy.

Considering the features listed above, the M-sequence radar sensors are already available in the commercial market and find a vast range of applications in almost all areas of industry and human life. Examples of the already published M-sequence radar deployments are listed below:

- Localization and positioning [24]
- Search and rescue [25,26,27,28]
- Ranging and collision avoidance [29]
- Geology and archaeology [30]
- Inspection of buildings [31]
- Non-destructive testing [32]
- Quality control [33]
- Metrology [34,35]
- Microwave imaging [36]
- Medical engineering [37,38,39]
- Law enforcement, intrusion detection [25,39]
- Assisted living [40,41]
- Industrial robot vision [42]
- Landmine detection [43].

## 3. 15th-Order M-Sequence Radar SoC

Recently, we have dealt with the integration of UWB radar head high-frequency blocks into a single integrated structure. It was necessary to solve the problem of integration of the transmitter, receiver, synchronization circuits, impedance matching of input ports, total power consumption, efficient placement of components on the chip, and many other tasks as far as the desired results were achieved. Integrated or monolithic SoCs have become a frequently discussed topic in many scientific papers and hardware manufacturers. In the case of hardware development, this is one of the most demanding processes. Modern progressive tools and methods of system integration or implementation of integrated structures, especially solutions of mixed analog–digital circuits, are continuously being improved by the development of new high-performance technologies. One such task is to design a new UWB-integrated radar based on a mixed structure in the form of an application-specific integrated circuit (ASIC). The most important radar parameters of the UWB are its operating bandwidth, which is very wide in comparison with devices that continuously emit electromagnetic waves, as well as the central frequency. Both of these parameters define the radar distance range and range resolution.

### 3.1. Monolithic Integration of UWB Radars

Since the semiconductor transistor was invented, there has been a lot of effort to minimize the transistor dimensions, increase reliability, and finally integrate several components on a single semiconductor substrate. The main reasons for that are economic and technological efficiency, which could not be achieved by discrete components. For the UWB short-range sensing technology, the main advantages of integration are as follows:

- Small size required by many industrial, medical, and commercial applications
- Higher reliability enabled by the elimination of connectors, cables, and soldering
- Simplified radio frequency (RF)and wideband design, because the on-chip circuit elements are much smaller compared to the wavelength; therefore, they can be considered as lumped elements
- High precision and stability thanks to good matching of components on a chip
- Low power consumption because of lower interconnect parasitics
- Availability of professional design software with good agreement of simulation and measurement results
- Low costs in mass production.

However, there are certain disadvantages of the integration of UWB radars, which have to be taken into account:

- High costs and turnaround time of prototype manufacturing and verification
- Difficult chip packaging maintaining good RF and UWB impedance matching
- Limited modifications and measurement of functionality once the chip is manufactured
- Higher costs of small volumes.

So far, there have been some UWB radars developed by research institutions as well as commercial companies that are based on application-specific integrated circuits (ASICs). The ASIC-based UWB radars are generally built either as a multi-chip or with an SoC architecture. Multiple UWB radars exploiting the ASIC technology are presented in Table 1 with their most important parameters.

From the above table, we can see that the most UWB radar SoCs are manufactured in Complementary MOS (CMOS) process and carry the pulse radar. The power consumption of CMOS radar systems is much lower compared to Bipolar CMOS (BiCMOS); however, both BiCMOS M-sequence radar systems are able to outperform the other radars shown in Table 1 in terms of bandwidth, range, and resolution. The integrated SoC radar whose performance was tested in this paper keeps the parameters of its multi-chip predecessor. Moreover, it features a much longer unambiguous range thanks to the increased M-sequence order.

In this section, we will deal with performance tests of the 15th-order M-sequence UWB radar SoC, whose circuit structure we published in detail recently [47]. However, the earlier description of this novel UWB sensor SoC was limited to the inner structure of the circuit and measurements of its basic electronic parameters only. In the following subsection, it will be compared to an earlier 9th-order multi-chip M-sequence device by output signal measurement as well as by practical deployment in a realistic measurement scenario.

### 3.2. 15th-Order M-Sequence Radar SoC Performance Measurements

We will first start with a description of the block diagram of the integrated structure of the UWB radar on one chip with the results of measuring its basic parameters directly on the die; then, we will introduce its implementation in the application for the measurement of the moving person through a long corridor (more than 40 m). The results from the measurements of two radars, the proposed 15th-order SoC and the conventional 9th-order multi-chip radar, were compared.

**Figure 4:**
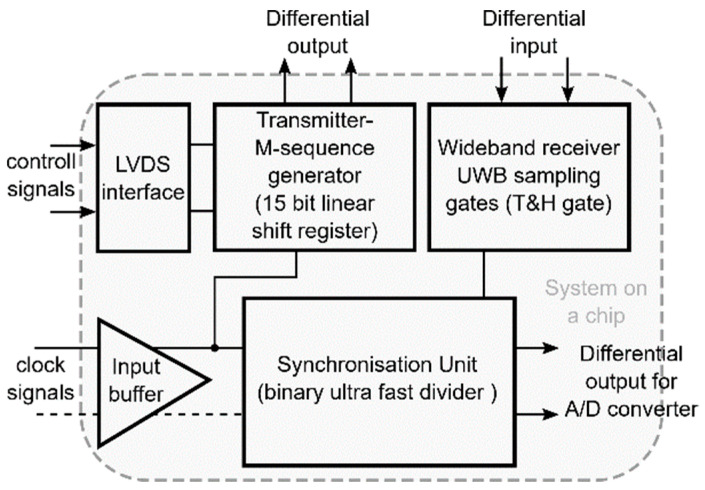
Integrated structure (SoC) diagram of the UWB radar.

The overall structure of the integrated analogue UWB radar head is depicted in the block diagram in Figure 4. The proposed and realized idea is based on the adapted and simplified conventional UWB radar concept shown in Figure 2. During the integration process, the transmitter circuits have been redesigned and replaced by the latest generation of the 15-bit M-sequence generator. The transmitter block was enhanced by the clock signal control as well. The main reason is to allow the operation in a multi-channel system and to interconnect the devices to create sensor networks in combination with other sensors [22]. As an example, applications requiring functionally safe non-optical high-resolution 3D sensing for object movement can be mentioned, which is designed for operation in unknown environments.

**Figure 5:**
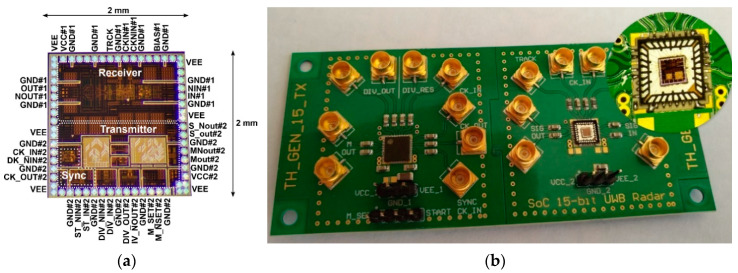
Integrated UWB radar SoC: (**a**) magnified view of the bare die and (**b**) evaluation module—transmitter (left) and receiver (right).

The proposed concept of the UWB radar implemented as SoC is built on the ASIC platform. This design was realized by using the 0.35 μm SiGe BiCMOS (S35D4) technology supplied by AMS, Austria. This process enables the realization of mixed analog–digital structures on a single chip and covers transit frequencies F_t_ up to 70 GHz. A photograph of the UWB radar SoC die is shown in Figure 5a. For extended measurements, the evaluation module was designed and manufactured (see Figure 5b), where the bare dies were packaged. The evaluation module uses two UWB radar SoCs—one as a receiver and the other as a transmitter. The values of the basic parameters of the proposed integrated structure such as clock frequency, overall power consumption, RF output power, and 1 dB compression point are nearly from DC up to 14 GHz, 1.6 W, (TX 790 mW and RX 810 mW), up to −7 dBm and around −7.9 dB, respectively. A more detailed description of these parameters as well as their measurement can be found in [48].

To evaluate the properties of the 15th-order M-sequence SoC radar, the spectrum of the transmitted signal was measured by a spectrum analyzer (Agilent MXA Signal Analyzer N9020A) and compared to the spectrum of the signal transmitted by another M-sequence radar equipped by a 9th-order M-sequence generator. Both radars were driven by the same 9 GHz clock signal.

**Figure 6:**
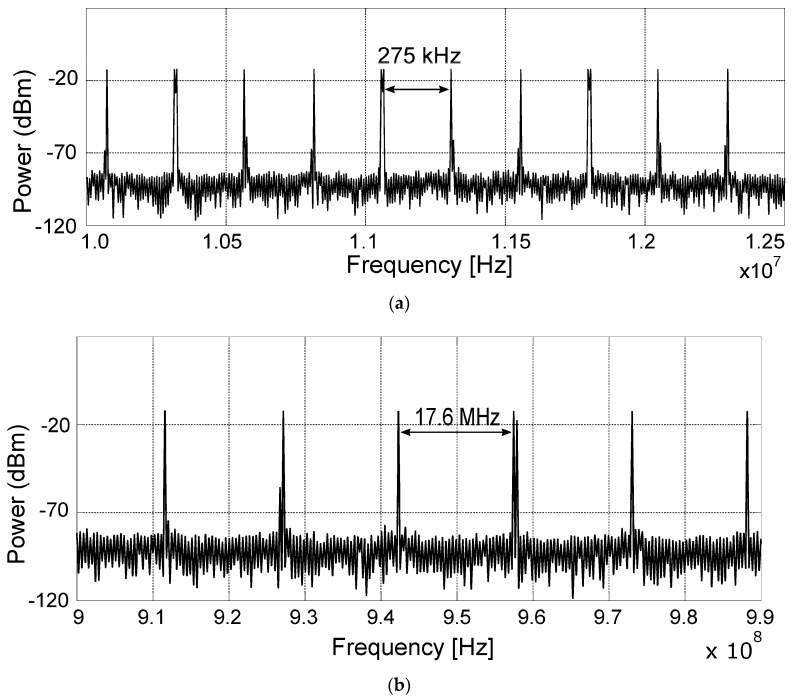
Comparison of spectral line spacing of signals transmitted by the 15th (**a**) and 9th (**b**) order M-sequence radars.

The main advantage of the 15th-order M-sequence radar over its lower-order versions is that it can use the spectral mask given by regulatory standards (either ECC or Federal Communications Commission (FCC) [15]), which are given in dBm/MHz more efficiently. The reason comes from the nature of the M-sequence signal and is illustrated by the spectrum measured in detail in Figure 6. The spectrum of the M-sequence has spectral lines spaced by

(1) $$\Delta f=f_{c}/(2^{m}-1)$$

where *f_c_* is the clock frequency and *m* is the order of the M-sequence. According to the Equation (1), the spectrum of the 15th-order M-sequence (Figure 6a) features spectral lines spaced by approximately 275 kHz, while the spacing for the 9th-order M-sequence is about 17.6 MHz (Figure 6b). Therefore, 3 or 4 spectral lines of the 15th-order M-sequence fall always into the 1 MHz frequency bin, but only zero to one spectral line of the 9th-order M-sequence falls into the 1 MHz segment given by regulations. Consequently, the proposed SoC should be able to radiate more total power while satisfying the regulatory conditions and possibly achieve better results in scenarios where targets at a large distance or high-attenuation environments are expected.

**Figure 7:**
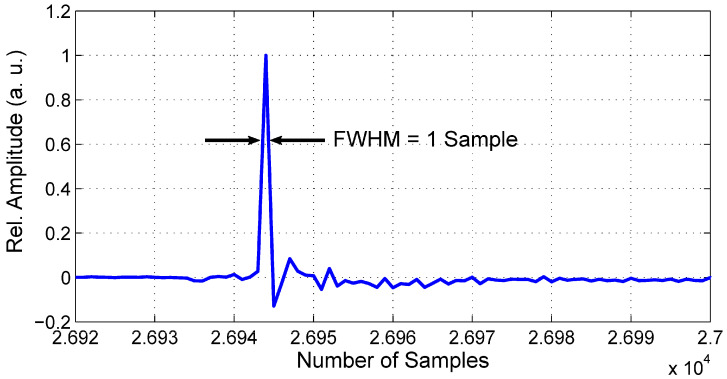
SoC closed-loop impulse response (pulse detail).

The spectrum of the signal radiated by the radar sensor gives us a picture about the radar sensor transmitter. On the other hand, the impulse response measured in the closed-loop configuration (transmitter connected to the receiver directly by a coaxial cable) provides the information about the quality of the receiver as well. In an ideal case, the received pulse should consist of a single non-zero sample. However, all system imperfections such as noise, signal reflections, crosstalk, clock jitter, and T&H transients come into account in reality. Nevertheless, the impulse response of the proposed SoC radar shown in Figure 7 has the full width at half maximum (FWHM) of 1 sample, corresponding with the theoretical assumption. The pulse is followed by a weak ringing caused by the mentioned system imperfections. Compared to previous systems that achieve an impulse response FWHM of about 2–3 samples, the obtained result is very good. As the impulse response has been measured at 9 GHz clock frequency, the FWHM of 1 sample corresponds to a pulse FWHM of 111 ps in time scale. The main improvement of the proposed SoC system impulse response can be assigned to a novel T&H circuit design, which allows for accurate adjustment of the sampling circuit by external biases.

**Figure 8:**
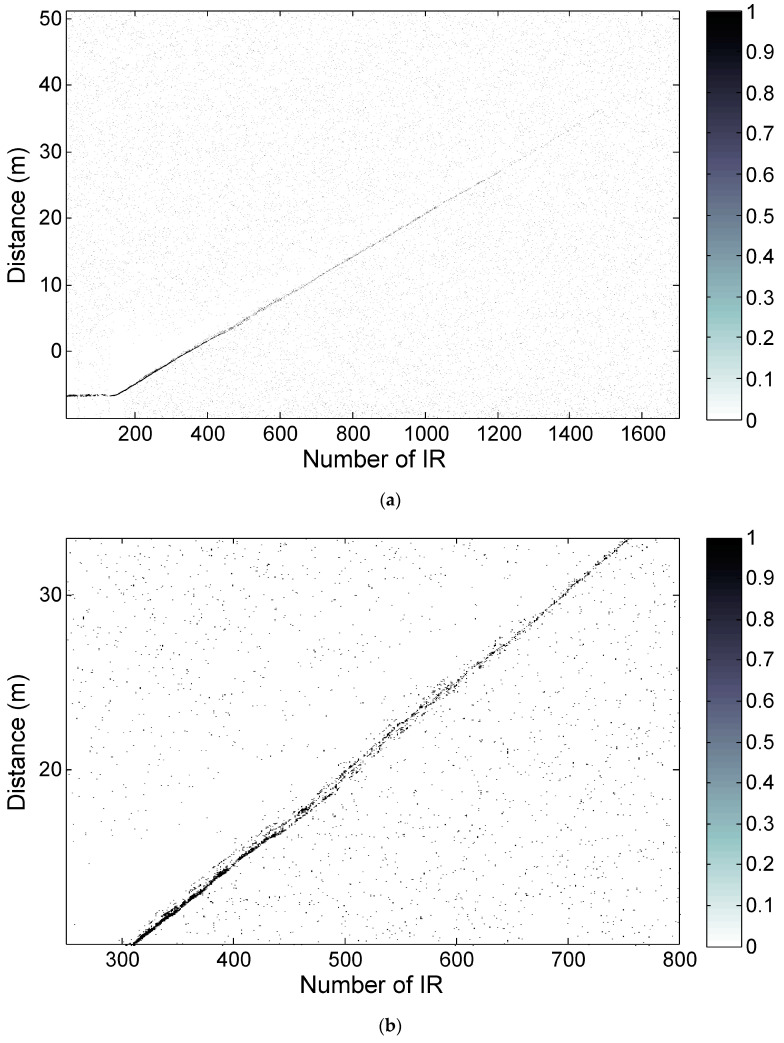
Detection of a moving person by 15th-order M-sequence SoC radar, maximum distance 41 m: (**a**) entire range of interest; (**b**) detail of trace fade-out at approximately 30 m.

**Figure 9:**
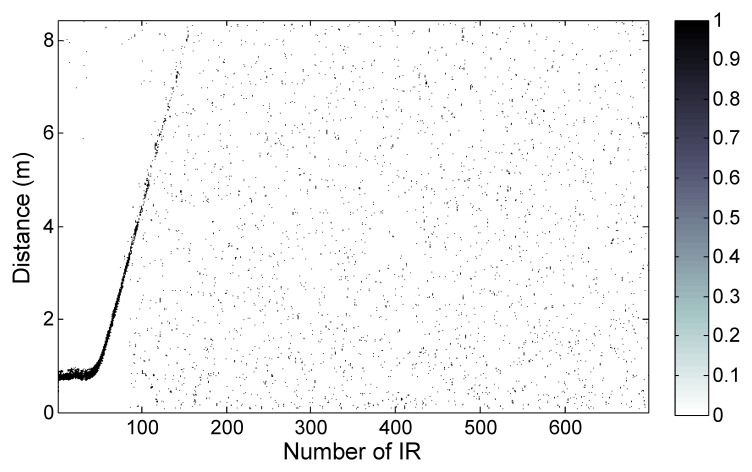
Detection of the moving person by 9th-order M-sequence multi-chip radar, maximum distance 41 m.

To compare the realistic performance of the proposed 15th-order M-sequence radar SoC to the earlier 9th-order device, we arranged a simple test scenario. The two M-sequence radars were placed at one end of the corridor in the university office building, while a person was walking down the corridor in a direction toward the other end of the corridor, which was 41 m away from the radar antennas. A moving person was observed by both radars, the proposed 15th-order SoC and the conventional 9th-order multi-chip radar, which were compared. It is important to note that the transmitter outputs of both radars were adjusted by attenuators to match the same spectral mask measured with 1 MHz resolution bandwidth, which was similar to the regulatory spectral masks mentioned earlier. The resulting radargrams are shown in Figure 8 and Figure 9.

The detection result of the moving person observed by the proposed SoC radar is shown in Figure 8. The continuous detection trace (white curve) of the person can be observed up to 10 m of range (Figure 8a); then, the increased density of detections (white points) is still distinguishable up to about 30 m (Figure 8b).

Figure 9 presents the detection results of the moving person scenario as captured by the 9th-order M-sequence radar. The target was detected continuously up to approximately 4 m and the increased density of detections is still apparent up to 10 m. Then, the target trace fades out totally. The radargram is wrapped by modulo 8.4 m because of the unambiguous range of the 9th-order M-sequence radar.

## 4. Integrated ECC Bandpass Filter for M-Sequence UWB Radar SoC

Analogue filters are important components of M-sequence UWB radar systems. On the transmitting side of the M-sequence radar, the main role of a filter is to ensure the compliance of the radiated signal with regulation requirements, mostly the ECC (Europe) or FCC (USA) rules [15,49]. In the receiving path of the UWB radar, the main roles of the filter are noise and aliasing suppression.

Methods to obtain the wanted spectrum of signals in both the transmitting and receiving path can be divided into two groups:

- the M-sequence signal can be filtered by a low-pass filter to the half of the desired bandwidth and mixed up to the center frequency of the desired band, resulting in two equal sidebands around the carrier.
- filtering the baseband M-sequence signal directly by a bandpass filter with the desired frequency response without additional frequency conversion.

For these two approaches, we experimentally designed appropriate active filters in the form of ASICs in the low-cost 0.35 µm SiGe BiCMOS technology. However, the design of filters on chip has several peculiarities, such as large variations of process parameters across the wafer, an unavailability of inductors, and a restricted range of capacitor and resistor values. The availability of amplifiers that could act as operational amplifiers in active filters is limited by the transit frequency of the given technology as well. These facts result in very limited possibilities to design filters with cutoff frequencies above 1 GHz in the given semiconductor technology. Therefore, the design of integrated filters was aimed rather at proving the viability of filter design in the given technology than at the design of a final device with superior characteristics. However, the improved versions of filters [50] can be designed later based on the knowledge gained from the proof-of-concept circuits. The design of an integrated low-pass filter with 1 GHz cutoff frequency has been described in detail in our earlier work [51]; therefore, we will discuss the bandpass filter design in this paper.

### 4.1. Circuit Structure with Negative Feedback

Negative feedback is a technique commonly used in broadband amplifiers. The main advantage of this technique is the reduction of the change in the behavior of the circuit due to changes in the parameters of its individual components, power supply, and temperature. Another key point is to improve input and output matchings. Signal distortion will also be reduced, and the bandwidth will be increased. However, the circuit gain is reduced, and the stability may be changed.

The most commonly used feedback topology is the resistance between the base and the collector of the first-stage transistor. However, this cannot achieve low noise, flat gain, and good input matching at the same time. Therefore, we used an original topology where the feedback is achieved by resistors and capacitance according to [52].

**Figure 10:**
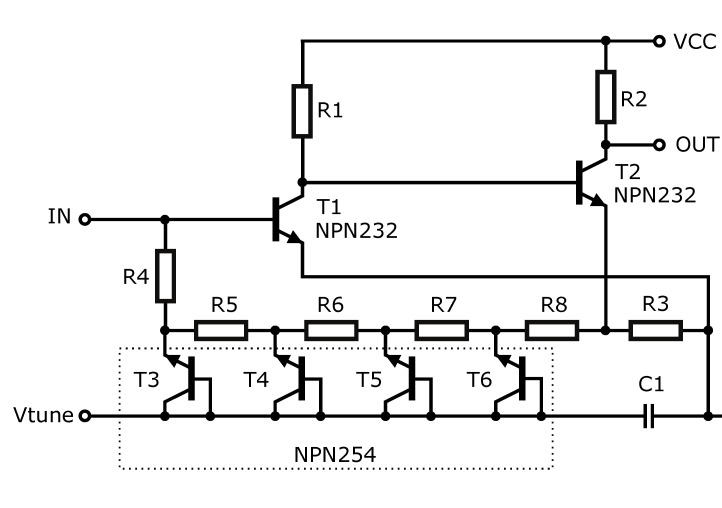
Simplified schematic of the proposed filter stage [53].

The circuit diagram of one stage of the proposed ECC bandpass filter is shown in Figure 10. This stage consists of two common emitter amplifier circuits with transistors T1 and T2. The emitter area of T1 is set for the best input matching and noise performance, while that of T2 is optimized for sufficient transit frequency. However, in the case introduced in [52], the feedback resistor was replaced by the RC low-pass ladder structure formed by resistors R4 to R8 and transistors T3 to T6. The reverse-polarized junctions of the transistor are used instead of capacitors to enable fine tuning of the filter by external DC voltage applied to port Vtune. The tuning capability is aimed at compensating for process variations, which may cause the filter characteristics to change slightly. Although the low-pass negative feedback should theoretically result in a high-pass behavior of the circuit, the gain of the amplifier is limited at high frequencies by the *f_t_* of transistors used. Therefore, a bandpass filter is obtained by a proper combination of these two phenomena.

Resistors R1 and R3 set the DC operating point of the amplifier stages to a value given by a trade-off between the optimal bandwidth and noise figure of transistors, taking the power consumption into account. The power consumption of one stage is 11 mA at a 3.3 V supply voltage. Resistor R1 sets the power consumption of the first stage as well as the input impedance at low frequencies. Resistor R3 is a key component of a circuit that sets many parameters, such as noise figure, input impedance, and also second-stage power consumption. Resistor R2 sets the output impedance to approximately 50 Ω. Resistors R4 to R8 must be high enough not to degrade noise performance. C1 capacity allows both the flat gain at higher frequencies and improves input matching. According to simulations, the resistance and capacitance values have been adjusted very carefully to ensure the stability of the circuit.

To achieve a better suppression of frequencies outside the passband, three stages with the same configuration as shown in Figure 10 are deployed in cascade. Moreover, additional RC high-pass elements are added in series with the active stages to isolate the DC biases apparent at the input and output nodes as well as to enhance the suppression of low frequencies.

**Figure 11:**
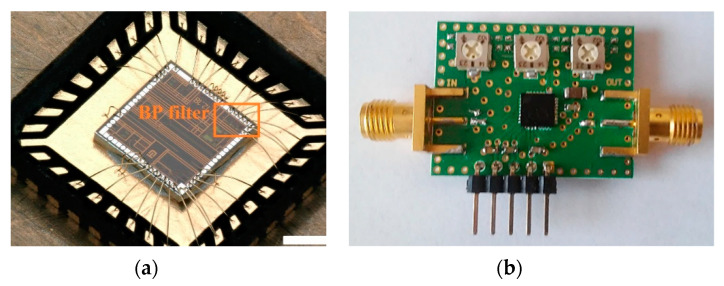
Bandpass filter ASIC prototype: (**a**) packaged die; (**b**) test printed circuit boards (PCB).

The proposed bandpass filter circuit was manufactured in 0.35 μm SiGe BiCMOS technology supplied by AMS Austria. To save prototyping costs, the filter was placed in a 2 × 2 mm die besides other circuits that are out of the scope of this paper. Firstly, the basic functionality was tested at the probe station. Then, the chip was packaged into a standard QFN32 package, and ports of selected circuits, including the proposed filter, were bonded to the package pins. A photo of the packaged die without a lid is shown in Figure 11a. The packaged IC was soldered to a test PCB equipped by SMA connectors for RF signals, trimmers for the adjustment of tuning voltages, and DC decoupling capacitors. The PCB is shown in Figure 11b.

### 4.2. Measurement Results

In this subsection, we will provide the S-parameter measurements of the filter prototype carried out at the probe station as well as on the test PCB. As a result of the limited number of available microprobes for testing at the probe station, only the tuning voltage of the first filter stage (V_t1_) was set to −2.8 V, while the other tuning inputs were left open. However, the test PCB allows for the setting of all three tuning voltages; therefore, the results for three tuning voltage sets are given to show the tuning abilities of the proposed filter. The voltage set (V_t1_, V_t2_, V_t3_) = −(1.8, 1.8, 1.8) V shifts the frequency response of the filter to the lowest frequencies, while (V_t1_, V_t2_, V_t3_) = −(3.8, 3.8, 3.8) V does the opposite. The best match with the ECC frequency regulation is achieved at (V_t1_, V_t2_, V_t3_) = −(4.15, 3.59, 4.0) V.

**Figure 12:**
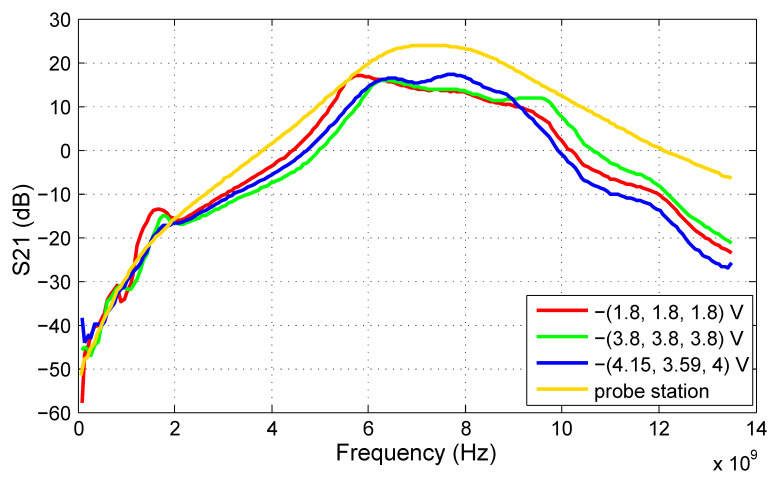
S21 of the proposed ECC bandpass filter.

The S21 results proving the functionality of the filter are shown in Figure 12. The 3 dB bandwidth of the filter spreads from 6 to 8.4 GHz for the best ECC match tuning. A difference between the measurements taken at the probe station and on the test PCB is apparent as well. The S21 measured directly on the die increases gradually with frequency if compared to the PCB measurements. The main cause can be the inductivity of bonding wires of the packaged IC.

**Figure 13:**
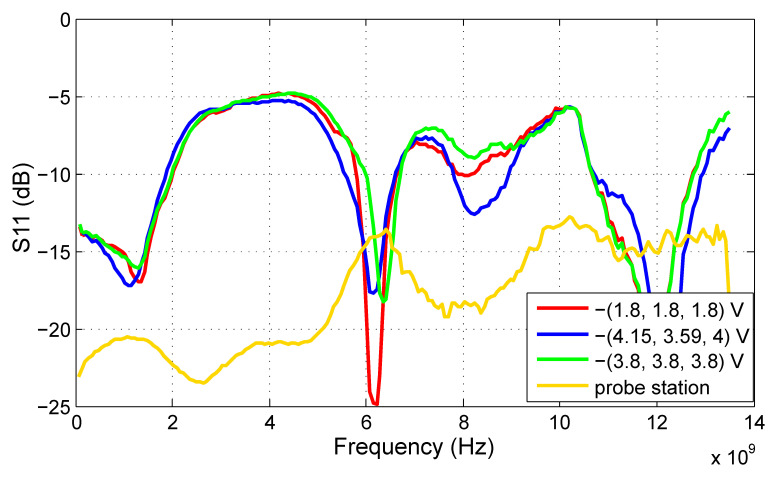
S11 of the proposed ECC bandpass filter.

**Figure 14:**
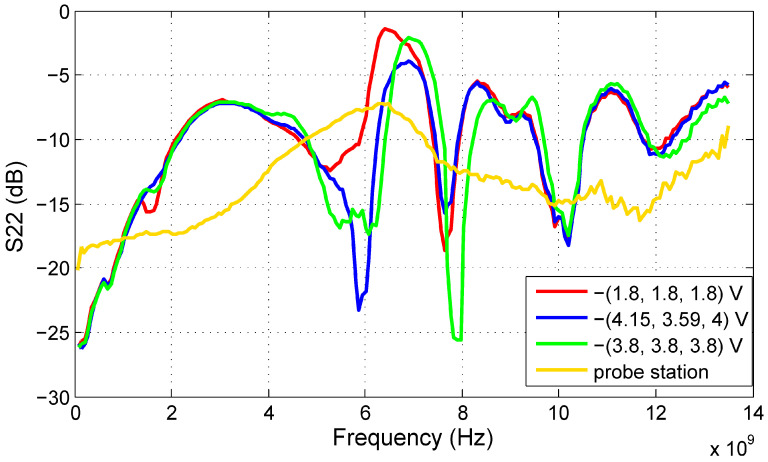
S22 of the proposed ECC bandpass filter.

The input and output reflection coefficients, S11 and S22 respectively, are shown in Figure 13 and Figure 14. Better results are achieved at the input, where the S11 values are below −7 dB within the passband of the filter. The output reflection coefficient of the filter should be improved in the future, because it reaches up to −2 dB under certain tuning conditions. However, the probe station results are significantly better than PCB measurements. Considering that in the final application, the proposed circuit should not be used as a stand-alone device but should be integrated on the chip with other components, the probe station results are more relevant. Moreover, the components on a chip are placed close to each other; therefore, the impedance matching is not so critical as for discrete components.

**Figure 15:**
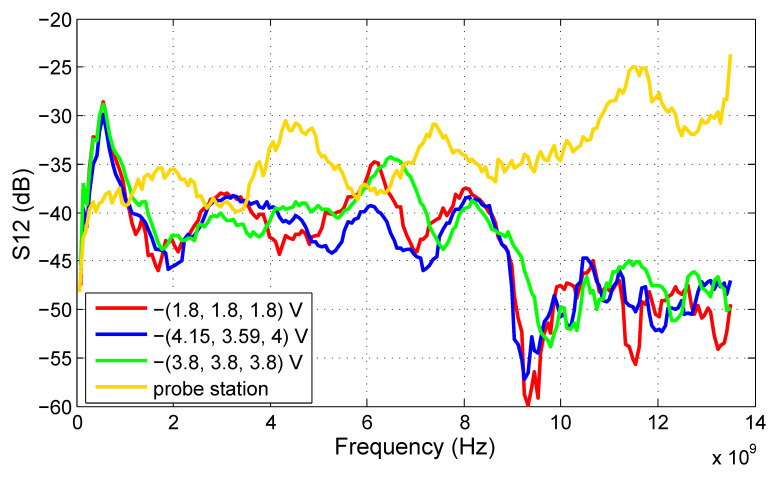
S12 of the proposed ECC bandpass filter.

The measurement results of the reverse transmission coefficient S12 are shown in Figure 15. The S12 is important for circuit stability. The obtained values for PCB measurements are well below −30 dB for the most of frequencies; therefore, they can be considered satisfactory. The S12 obtained by probe station measurements increases with frequency. However, this is a common behavior for this measurement method and is probably caused by the design of the microprobes and their placement close to each other.

## 5. Miniature UWB Transmitting Antenna for M-Sequence Radars

After the feasibility of the concept of electrically short active antennas for M-sequence UWB radars was confirmed by the experiments with discrete components in our previous work [54], customized ASIC circuits were proposed. The receiving short dipole UWB antenna for M-sequence radar application together with its ASIC amplifier design was proposed in [55]. The design of the transmitting antenna driver ASIC, as well as the design of a miniature active antenna exploiting it, will be shown in this section. Its functionality will be confirmed by experimental results.

### 5.1. Antenna Driver ASIC

**Figure 16:**
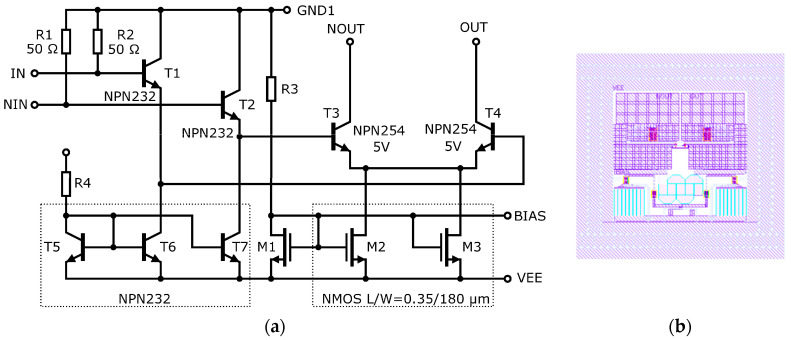
Proposed LCR driver circuit: (**a**) schematic diagram; (**b**) layout.

In this subsection, we propose a circuit designed for driving the transmitting Large current radiator (LCR) antenna by a sufficient RF current. As the topology to accomplish this task, a basic differential amplifier with open collector output has been chosen. The simplified schematic diagram of the proposed circuit is shown in Figure 16a. The proposed LCR driver has two stages—the input buffer and the output stage. The input differential RF signal is fed into the differential input (IN and NIN). The inputs are internally matched to 50 Ω single-ended impedance by resistors. The input buffer of the LCR driver circuit is made of emitter followers, which mitigate the influence of the output stage on the input of the circuit. The output stage of the LCR driver is formed by a differential pair consisting of two NPN254h5 transistors featuring the highest possible emitter area of 96 µm^2^. Despite the worse RF performance of the selected transistors, they have been chosen to accommodate the need for a robust amplifier output capable of delivering a maximum current of more than 100 mA at voltages above 5 V. Those parameters are better suited for relatively large radiators (more than 1 cm length), which can be efficient at frequencies below 5 GHz. For improved performance at higher frequencies, another driver circuit can be designed later, sacrificing some of the power capabilities, using smaller and faster 3.3 V transistors, or exploiting a different technology with higher f_t_ in general.

The total DC current of the power stage is set by a current mirror consisting of a reference transistor MN2 and two paralleled NMOS transistors, MN0 and MN1. The NMOS transistors are used because of their ability to build up a current mirror with a higher ratio of transistor areas, therefore allowing for lower reference current and saving power. The quiescent current of the output stage can be adjusted externally by the BIAS port. The resistance and width of the current mirror reference resistor allow for shorting the BIAS port to VEE; therefore, it is possible to turn the LCR driver off. This function can be beneficial in some applications, especially those battery-supplied. The input stage of the proposed LCR driver consumes 30 mA from a −3.3 V power supply attached between the GND1 and VEE ports. The positive pole of the output stage power supply should be connected to the LCR antenna. The circuit was designed to operate at a nominal DC voltage of +1.7 V at the OUT and NOUT outputs if referred to GND1 (+5 V if referred to VEE). The total DC current of the output stage is set to 100 mA if BIAS is left open. However, it can be adjusted between 0 and 200 mA. The outputs can be DC connected to GND1 as well; in that case, the default DC current is about 60 mA.

The layout of the LCR driver cell is shown in Figure 16b. The LCR driver cell requires a chip area of 470 × 480 µm. Most of the area is occupied by metallic interconnects of power supply and output ports to ensure low-impedance paths for relatively high RF currents and satisfy the requirements for the maximum current densities of metallic layers with a reasonable margin. The layout is symmetrical in accordance with the differential nature of the circuit. The layout of the cell was placed on a 2 × 2 mm die together with other ground-independent circuits to save prototyping costs. Multiple capacitors have been used to fill up the remaining chip area and RF-bypass the power supply rails.

Before submitting the circuit layout to the foundry, multiple simulations have been performed to check and adjust its performance. However, because of the open collector output of the proposed circuit, common S-parameter simulations have been difficult to perform and evaluate. For this reason, we simulated transient analysis. The circuit was stimulated by a differential rectangular voltage source with a rise time of 1 ps and voltage of 1 Vpp. The amplitude and the rise, as well as fall time of the output current at ports OUT and NOUT, have been observed while they were attached directly to the +1.7 V DC voltage source (referred to as GND1). The maximum current amplitude was 100 mA with the rise time of 112 ps and fall time about 87 ps. From the rise time, we can calculate the amplifier bandwidth according to:

(2) $$B=0.35/\tau_{r}$$

If we take the rise time *τ_r_* = 112 ps, we obtain the 3 dB bandwidth of 3.125 GHz, which is sufficient, especially for applications where the miniaturization of antennas was difficult so far because of the low operational frequency.

**Figure 17:**
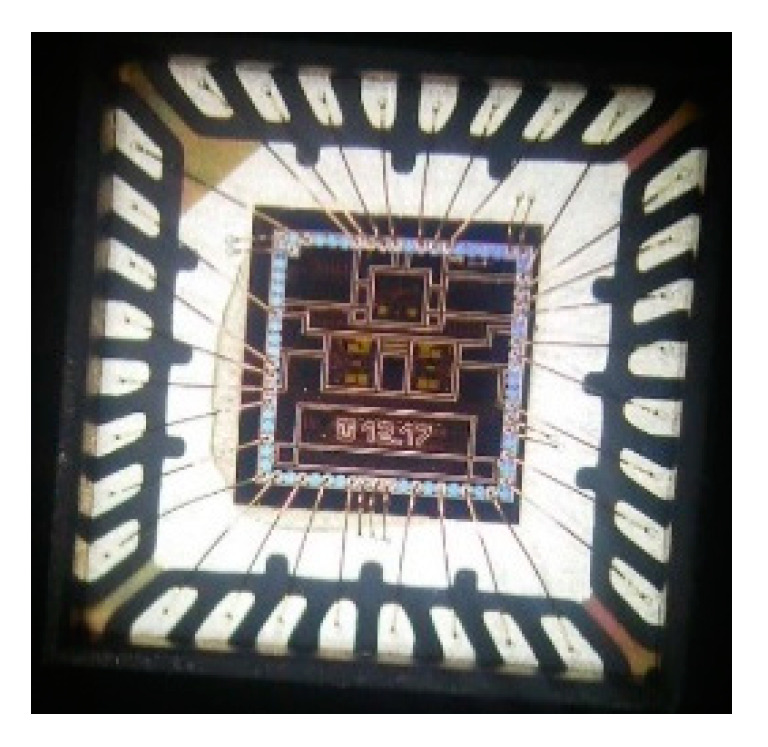
Prototype of the chip carrying the proposed LCR driver circuit in QFN32 package.

After testing at the probe station, the prototype die was packaged into a standard QFN32 package, as shown in Figure 17. The LCR driver output bonds (OUT, NOUT), as well as the ground connections (GND1), are doubled to reduce the inductance of the bonding wires.

**Figure 18:**
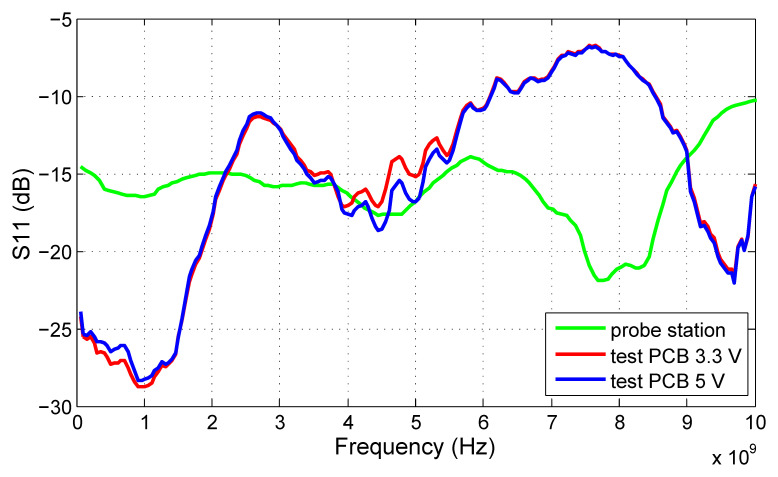
S11 of the proposed LCR driver measured at the probe station (green) on the test PCB with 3.3 V output stage power supply (red) and with 5 V output stage power supply (blue).

Although the most relevant testing of the proposed LCR driver will be performed together with the radiator element, measurements have been performed on the test PCB as well. Firstly, the reflection coefficient S11 of the input ports IN and NIN was measured by a network analyzer. The same has been done earlier at the probe station, and the comparison of the results obtained from the packaged IC and the probe station measurements is shown in Figure 18. Moreover, the influence of the output stage power supply is observed by the comparison of the curves acquired from the measurements at 3.3 V (referred to VEE, outputs connected to GND1) and 5 V (1.7 V if referred to GND1). From the figure, it is obvious that although the final stage does not influence the input impedance of the amplifier, the measurement method makes a significant difference. Although the probe station measurement technique can introduce notable contact resistance that is in series with the real DUT impedance, the measurements on the test PCB include bond inductance, package parasitics, and PCB trace impedance mismatch while minimizing ohmic losses in the measurement setup. This is confirmed by the flat curve acquired from the probe station corresponding to the wideband resistive impedance, whereas the S11 measured on the PCB varies significantly across the frequency, confirming the higher reactive component of the input impedance. However, all S11 measurement results are below −10 dB up to 6 GHz, which is a satisfactory result for the proposed circuit. The S11 of both IN and NIN ports is almost the same; therefore, only results for the IN port are shown in Figure 18.

**Figure 19:**
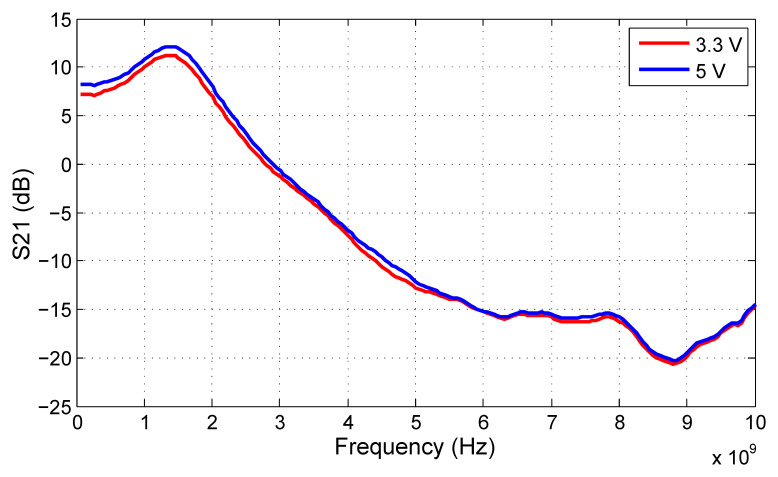
S21 of the proposed LCR driver circuit measured with 10 Ω dummy load.

The gain of the proposed LCR driver circuit can be measured by a Vector Network Analyzer (VNA) in terms of an S21 forward transmission coefficient to illustrate its gain and bandwidth behavior. However, the loading impedance of the output ports has to be specified in this case. For this measurement, we have chosen to use two 10 Ω resistors connected to the open collector ports as dummy loads instead of an LCR antenna. The resulting S21 chart is shown in Figure 19. Again, we compare the influence of the supply voltage of the output stage by using 3.3 V and 5 V power supply referred to VEE. However, the supply voltage has minor influence on S21. The 3 dB bandwidth of the circuit loaded by 10 Ω is about 2 GHz, while the 10 dB bandwidth reaches 3 GHz. However, the real performance of the LCR driver circuit depends heavily on the loading impedance, which will be given by the radiator design; therefore, these measurement results should be considered illustrative.

### 5.2. LCR Antenna Measurement Results

To prove the functionality of the active circuit for the electrically short transmitting antenna described above, an example of an active antenna design was proposed and tested. It is the miniature LCR transmit antenna operating at frequencies from 160 MHz to 2.4 GHz. Although this frequency range is not conforming with the ECC nor FCC specifications, the antenna can be used for wall or ground-probing applications, where the regulations apply only to signals radiated out of the tested material.

**Figure 20:**
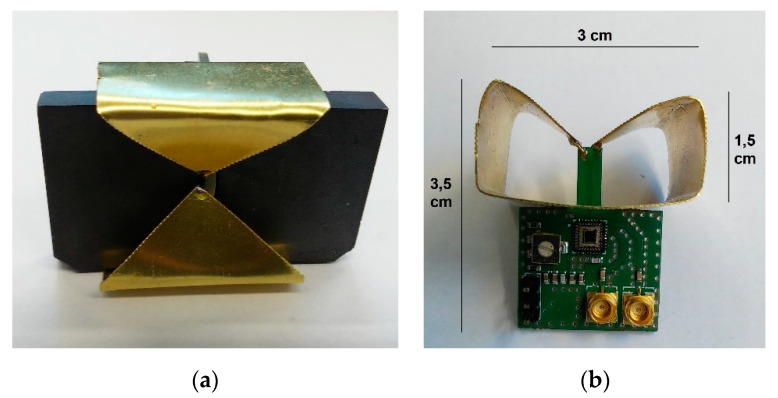
Mechanical setup of the proposed LCR antenna: (**a**) front view, (**b**) side view without ferrite absorber tiles.

The mechanical setup of the proposed antenna is shown in Figure 20. The self-grounded bowtie shape was chosen for the radiator design. The radiator dimensions are 3 × 3 × 1.5 cm, to correspond with the intended operation frequencies below 3 GHz. The tips of the self-grounded bowtie are soldered to the PCB protrusion with differential paths connected to the driver outputs, while the back side of the radiator is soldered to a wide VCC pad on the bottom layer of the PCB. Two pieces of ferrite absorber tile C-RAM FT-10 [56] are placed inside the radiator. The PCB with driver electronics is not shielded because of the difficult ferrite cutting process. However, experiments showed only minor opportunities for improvements by shielding the whole PCB, while it would increase the weight and cost of the antenna significantly.

**Figure 21:**
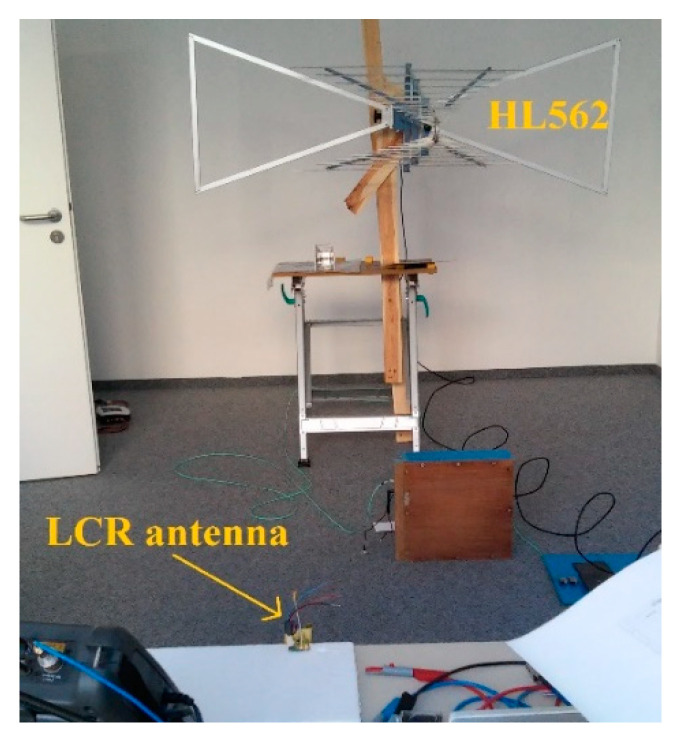
Measurement setup for the proposed miniature LCR antenna for wall and ground-probing applications.

The transmitting LCR antenna prototype was measured in the setup shown in Figure 21. The transmission coefficient of two antennas has been measured by a VNA. As the receiving antenna, a Rhode & Schwarz Ultralog HL562 was used for its good performance at frequencies from 30 MHz to 3 GHz [57]. The distance between the feed points of the antennas was 3 m. The measurement was carried out in a laboratory without anechoic treatment.

**Figure 22:**
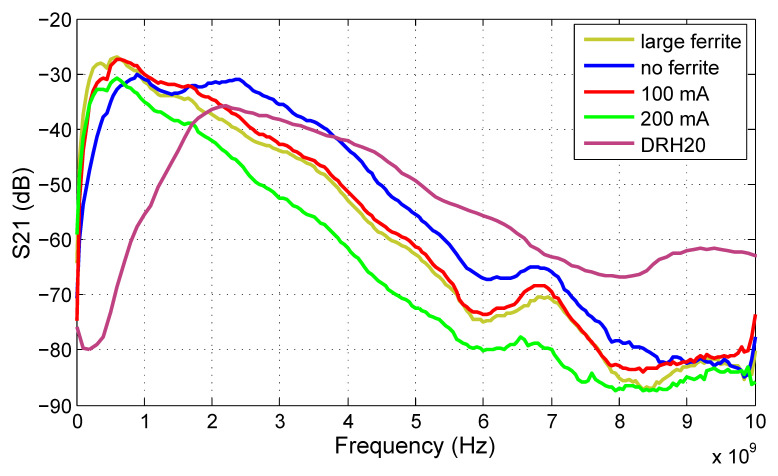
Transmission coefficient of the proposed LCR antenna measured under multiple different conditions: yellow—with larger absorbers, blue—without absorber, red—100 mA output stage current, green—200 mA output stage current. Professional antenna DRH20 measured for comparison (purple).

The results of transmission coefficient measurements are shown in Figure 22. The proposed antenna was measured under multiple different conditions to illustrate its behavior [58]. The configuration shown in Figure 20a was tested at two output stage current levels 100 and 200 mA at +3.6 V VCC voltage (referred to the ground) applied to the radiator. Surprisingly, the antenna operates better at lower current, although a minor improvement at low frequencies below 200 MHz is achieved. The main reasons can be the saturation of the output stage by high current. The red curve corresponding to the 100 mA current can be considered as the nominal result achieved by the proposed antenna. The 10 dB bandwidth reaches from 160 MHz to 2.4 GHz. Although the absolute bandwidth of the antenna is only 2.24 GHz, the fractional bandwidth of the antenna is 187%, and the antenna is able to efficiently transmit a signal even at frequencies where the wavelength is more than 50 times greater than the antenna size.

Additionally, the antenna was tested without a ferrite absorber and with an absorber; the size increased to about 10 × 2.5 cm in total. The results prove that the absorber material significantly lowers the lower cutoff frequency of the antenna. However, the upper cutoff frequency rises to almost 4 GHz without the absorber. From this point of view, the same antenna can be used with absorbers for lower bands as well as without absorbers for higher frequencies, depending on the application. For better comparison, the professional DRH20 antenna [59] was measured under the same conditions. From the comparison, it is clear that a significantly bigger professional antenna has been outperformed by the proposed antenna in terms of low-frequency operation.

## 6. Conclusions

It is obvious that the technology acceptance goes hand in hand with the ability of the technology to solve broader application tasks using one hardware family on one side, and with the possible lowest price level on the other. The constant research on extensions of the existing family of M-sequence-based UWB hardware components, including both core system components [15] such as new versions of stimulus generators and application-specific system front-ends, such as antenna drivers or filters, is therefore a logical consequence. This work described the designs of multiple M-sequence UWB radar front-end subcircuits realized in the form of application-specific integrated circuits to enable the integration of front-end electronics with the novel M-sequence radar core available from previous research activities. This way, a new system on a chip (SoC) can be formed in the future with minimum external components required. Based on the previous work, the aim of the manuscript has been set, and recent research results were presented. Specifically in this paper, we described three new components for the extension of this family designed and realized on a piece of silicone. These are the integrated 15th-order M-sequence transceiver chip, active ECC bandpass filter, and miniature transmitting UWB antenna with an integrated amplifier. The proposed components, together with the components described in our previous works, form a solid background for the next steps in the enhancements, e.g., miniaturization of the M-sequence-based sensor technology and thus broadening of the application field.

The experimental results show that the proposed 15th-order SoC M-sequence radar achieves in some characteristics better results if compared to the 9th-order M-sequence radar. In addition to the increased unambiguous range given by the M-sequence order at the given clock frequency, the 15th-order M-sequence signal has more spectral components and therefore is able to exploit the available frequency spectrum more efficiently. Moreover, the closed-loop impulse response of the SoC radar features a full width at half maximum of only a single sample, which is superior to older M-sequence radars available in our lab, achieving the FWHM between 2 and 4 samples. This feature adds to the increased spectral efficiency and enables enhanced target detection. However, the impulse response of the 15th-order M-sequence radar is relatively long and causes limitations of measurement speed and/or averaging factor. Although it is possible to increase the signal-to-noise ratio by a higher averaging factor in 9th-order M-sequence radar, the proposed SoC radar seems to have superior properties even if operated at the same measurement speed. Therefore, it is much better suited for applications where targets are to be observed at long ranges or behind obstacles causing high attenuation.

The integrated ECC bandpass filter prototype for M-sequence UWB radars featuring a passband from 6 to 8.4 GHz was successfully designed, fabricated, and measured as well. The viability of the RF filter design in cost-efficient technology has been proved. The filter exploits a novel topology based on the RC network in the feedback of a low-noise amplifier. The filter features almost 20 dB amplification in the passband, making it suitable for applications at the receiver input. However, its main disadvantages are the limited dynamic range and insufficient output matching. The filter features the possibility of tuning by external DC voltages, which is aimed at compensating for deviations caused by process variations.

As the last component in this paper, the active transmitting antenna was proposed. From the comparison with a standard horn antenna, it is clear that a significantly bigger professional antenna has been outperformed by the proposed antenna in terms of low-frequency operation. This was achieved thanks to the UWB ASIC amplifier integrated into the antenna structure, compensating for small radiator inefficiency and mitigating impedance-matching problems. The volume of the antenna was reduced by a factor of 30 if compared to the reference antenna, while the lower cutoff frequency was reduced down to 160 MHz with all antenna dimensions below 3.5 cm. The dimensions and operational bandwidth of the antenna make it suitable for handheld material-penetrating devices such as through-wall and ground penetrating radars. However, the antenna properties were examined only by comparison to another reference antenna. It would be beneficial to measure the transmitting antenna properties with more appropriate equipment in a UWB anechoic chamber to obtain accurate values of antenna characteristics such as the gain, directivity, efficiency, etc.

The results of the presented research can be considered as a significant step toward a fully integrated M-sequence radar, including all components from the AD converter to the antenna ports on a single chip. This option is enabled by the design of all components in the same 0.35 μm SiGe BiCMOS technology, which is a cost-efficient solution for this task. However, such a complex integration will require further research work.

## Figures and Tables

**Table 1:** Overview of application-specific integrated circuit (ASIC)-based ultra-wideband sensors (UWB) radars. SoC: system on chip.

Manufacturer, Type, Reference	Ilmsens, GmbH M:Explore SH-3100 [17]	Novelda Xethru X4 [44]	Umain HST-C1R [45]	Zito, D. et al. [46]	This Work
**architecture**	multi-chip ** and SoC ***	SoC	SoC	SoC	SoC
**sounding signal**	M-sequence	pulse	pulse	pulse	M-sequence
**bandwidth**	up to 6 GHz,	1.4–1.5 GHz (−10 dB)	1 GHz	2 GHz	up to 5 GHz
**center frequency**	up to 3 GHz	7.29–8.748 GHz	3–5 GHz	3.5 GHz	up to 2.5 GHz
**range resolution**	>17.6 mm	10–11 cm *	15 cm *	7.5 cm *	3 cm *
**max. range**	up to 120 m	25 m	11 m	-	>980 m
**IC technology**	SiGe 0.35 μm BiCMOS	CMOS	130 nm CMOS	90 nm CMOS	SiGe 0.35 μm BiCMOS
**components included on chip(s)**	M-sequence generator, clock buffers, binary divider, track & hold, synchronization	transmitter, receiver, ADC, clock management, system controller	transmitter, receiver, ADC	pulse generator, shaper, delay generator, LNA, mixer, integrator	M-sequence generator, clock buffers, binary divider, track & hold, synchronization
**power consumption**	1.7 W	119 mW	100 mW	73.2 mW	1.6 W

* calculated from bandwidth, ** general purpose devices, *** application specific devices.
